# The dataset from administration of single or combined immunomodulation agents to modulate anti-FVIII antibody responses in *FVIII* plasmid or protein primed hemophilia A mice

**DOI:** 10.1016/j.dib.2016.03.019

**Published:** 2016-03-17

**Authors:** Chao Lien Liu, Meghan J. Lyle, Simon C. Shin, Carol H. Miao

**Affiliations:** a Center for Immunity and Immunotherapies, Seattle Children׳s Research Institute, Seattle, WA, USA; b School of Medical Laboratory Science and Biotechnology, College of Medical Science and Technology, Taipei Medical University, Taipei, Taiwan; c Department of Pediatrics, University of Washington, Seattle, WA, USA

## Abstract

Hemophilia A mice with pre-existing inhibitory antibodies against factor VIII (FVIII) were treated with single agents, AMD3100 and GCS-F, respectively. Inhibitor titers in treated mice and control HemA inhibitors mice were followed over time. Total B cells and plasma cells (PCs) were characterized by flow cytometry. HemA inhibitor mice were then treated with a combination regimen of IL-2/IL-2mAb complexes plus rapamycin and AMD3100. Finally, HemA inhibitor mice were treated with a new combination therapy using include IL-2/IL-2mAb complexes + Anti-CD20+AMD3100+G-CSF. The timeline of combination therapy was illustrated. Inhibitor titers following treatment in *FVIII* plasmid or protein induced inhibitor mice were evaluated overtime. A representative figure and gating strategies to characterize the subsets of Treg cells and B cells are presented. Please see http://dx.doi.org/10.1016/j.cellimm.2016.01.005 [1] for interpretation and discussion of these data and results.

## **Specification Table**

**Table:** 

Subject area	*Immunology*
More specific subject area	*Immunotherapy*
Type of data	*Figures*
How data was acquired	*Animal experiments, flow cytometry (*LSRII flow cytometer (Becton Dickinson, Palo Alto, CA)
Data format	*Analyzed*
Experimental factors	*Animals were immunized to generate inhibitory antibodies*
Experimental features	*Groups of animals were treated with plasmid mediated gene therapy or protein replacement therapy followed by immunomodulation*
Data source location	*Seattle, USA*
Data accessibility	*Data is with this article*

## **Value of the data**

• This is the first attempt to use stem cell mobilizing agents to modulate pre-existing anti-FVIII antibodies.
• Using combination therapy with Treg expansion agents IL-2/IL-2mAb complexes and rapamycin illustrates the importance of Treg cells not only in prevention of anti-FVIII antibody production but also in modulating pre-existing antibody responses.
• Detailed analysis of changes in subsets of T and B cells can help elucidate the mechanisms of these immunomodulation agents with single or combination treatment.

## 1 Data

**Fig. 1:**
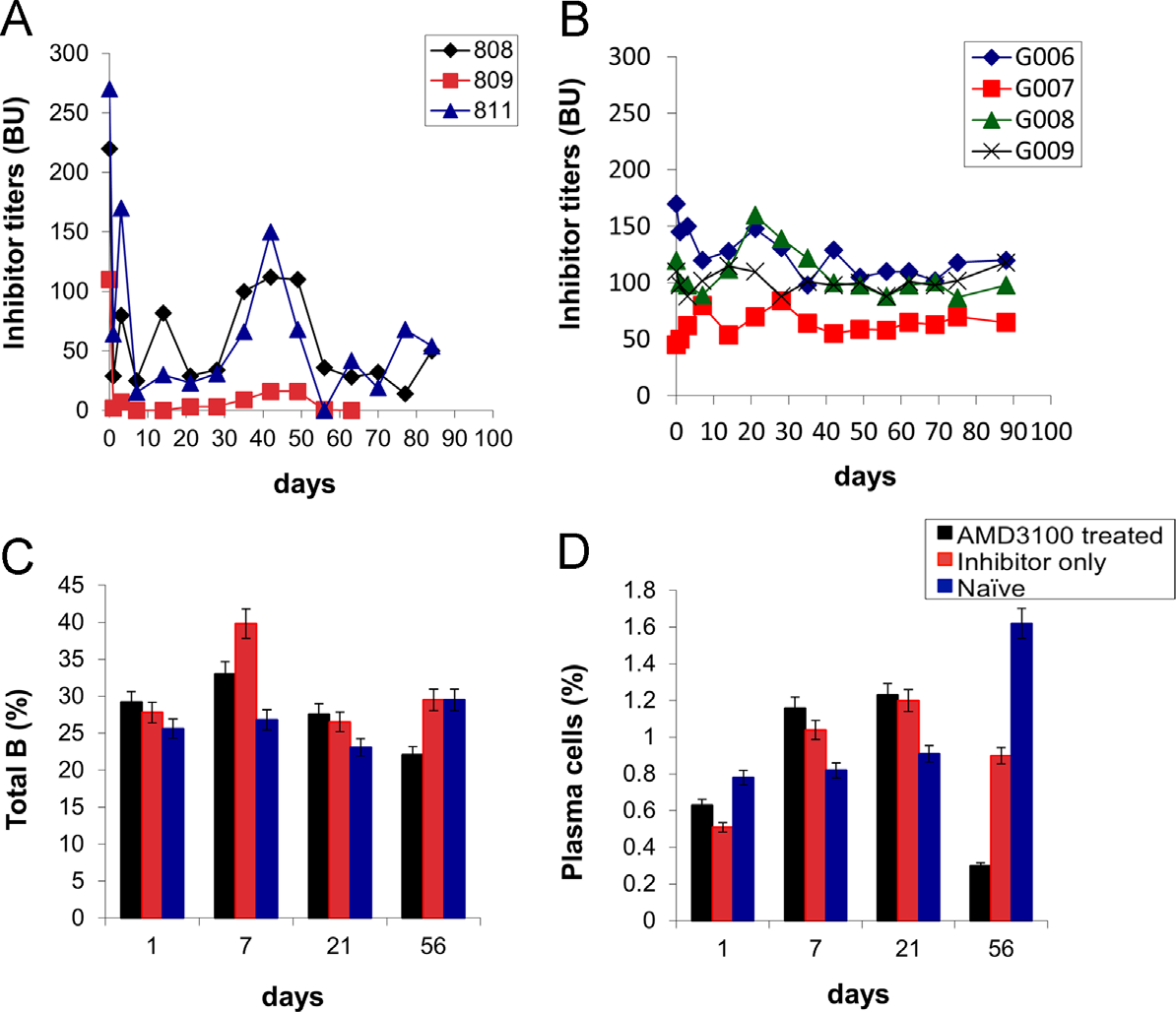
Single cycle of AMD3100 treatment reduced inhibitor titers in HemA inhibitor mice. HemA inhibitor mice were treated with 200 μg/mouse/day for 10 days. Significant reduction of titers were observed in treated mice (A; *n*=3), compared to the inhibitor only controls (B; *n*=4). (C and D) showed B cells staining results. Total B (C) and PCs (D) were decreased in the AMD3100 treated mice. Data shown is representative of two independent experiments.

**Fig. 2:**
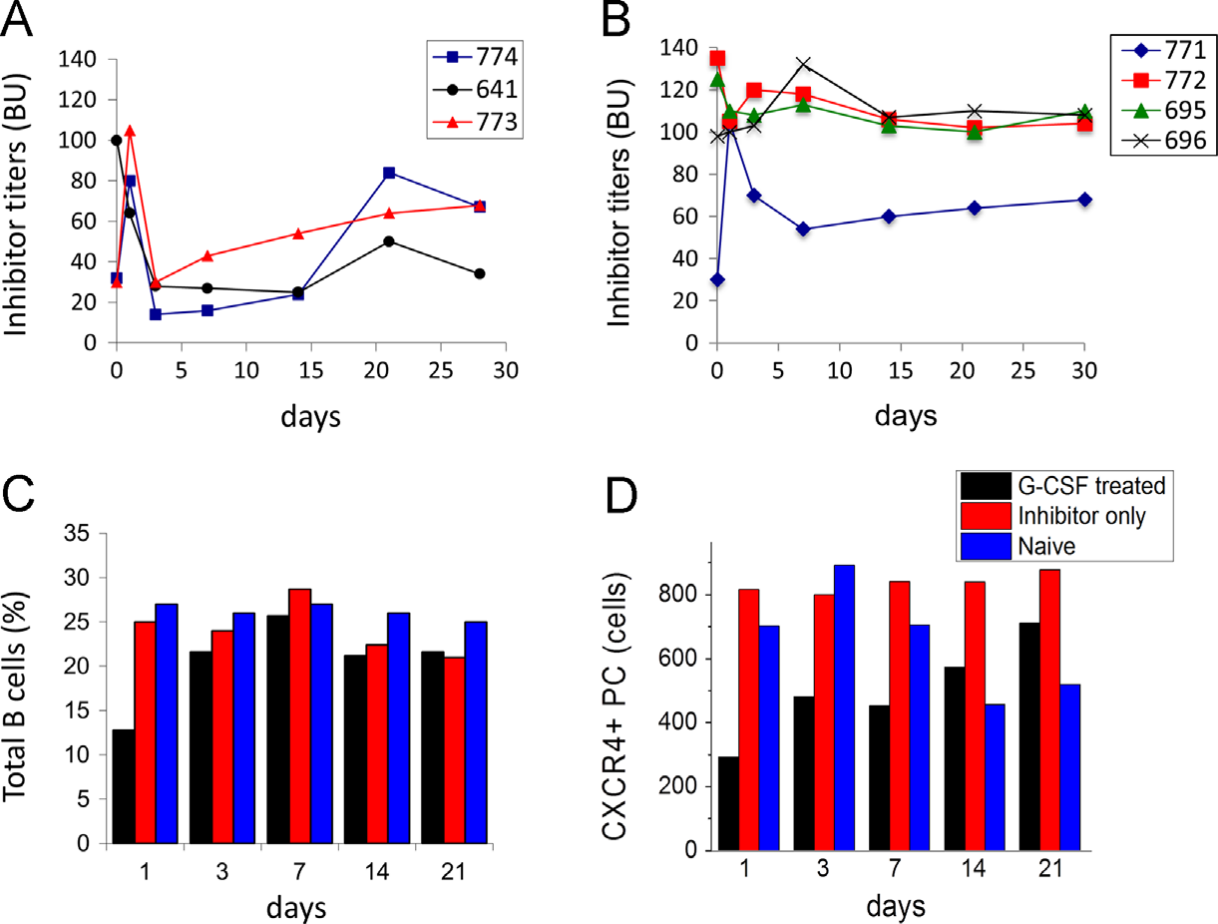
Single cycle of G-CSF treatment transiently reduced the inhibitor titers in HemA inhibitor mice. HemA inhibitor mice were treated with G-CSF 250 μg/kg/day for 5-days. The inhibitory titers were transiently reduced in G-CSF treated mice (*n*=3) (A), whereas high levels of inhibitors titers persisted in inhibitor only control mice (*n*=4) (B). B cell staining results showed that total B (C) and CXCR4^+^ PCs (D) were slightly reduced after the G-CSF treatment. Data shown is representative of two independent experiments.

**Fig. 3:**
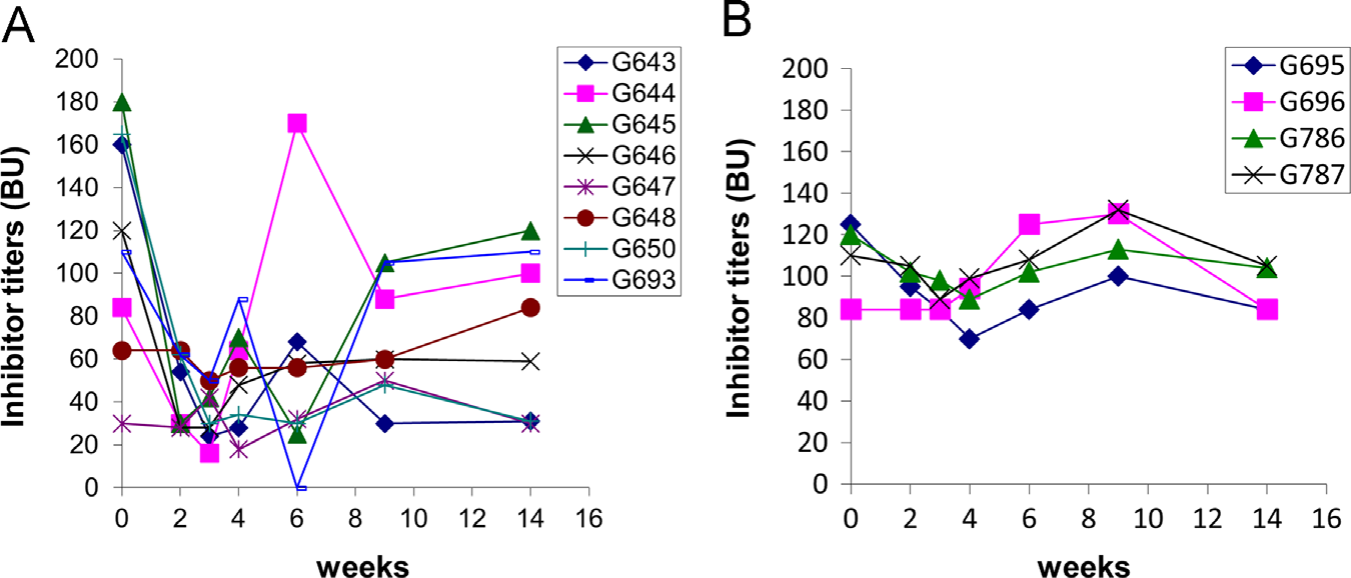
Inhibitor titers were reduced during the treatment with IL-2/IL-2mAb complexes+rapamycin+AMD3100 in *FVIII-*plasmid treated HemA mice with pre-existing inhibitors. *FVIII* primed inhibitor mice (*n*=8) were treated with IL-2/IL-2mAb complexes+rapamycin+AMD3100 for 4 weeks. (A) The inhibitor titers were reduced during the treatment period and sustained for 2 more weeks, however the titers returned to higher levels afterwards compared to persistently high levels of inhibitor titers in the inhibitor only mice (*n*=4) (B). Data shown is representative of two independent experiments.

**Fig. 4:**
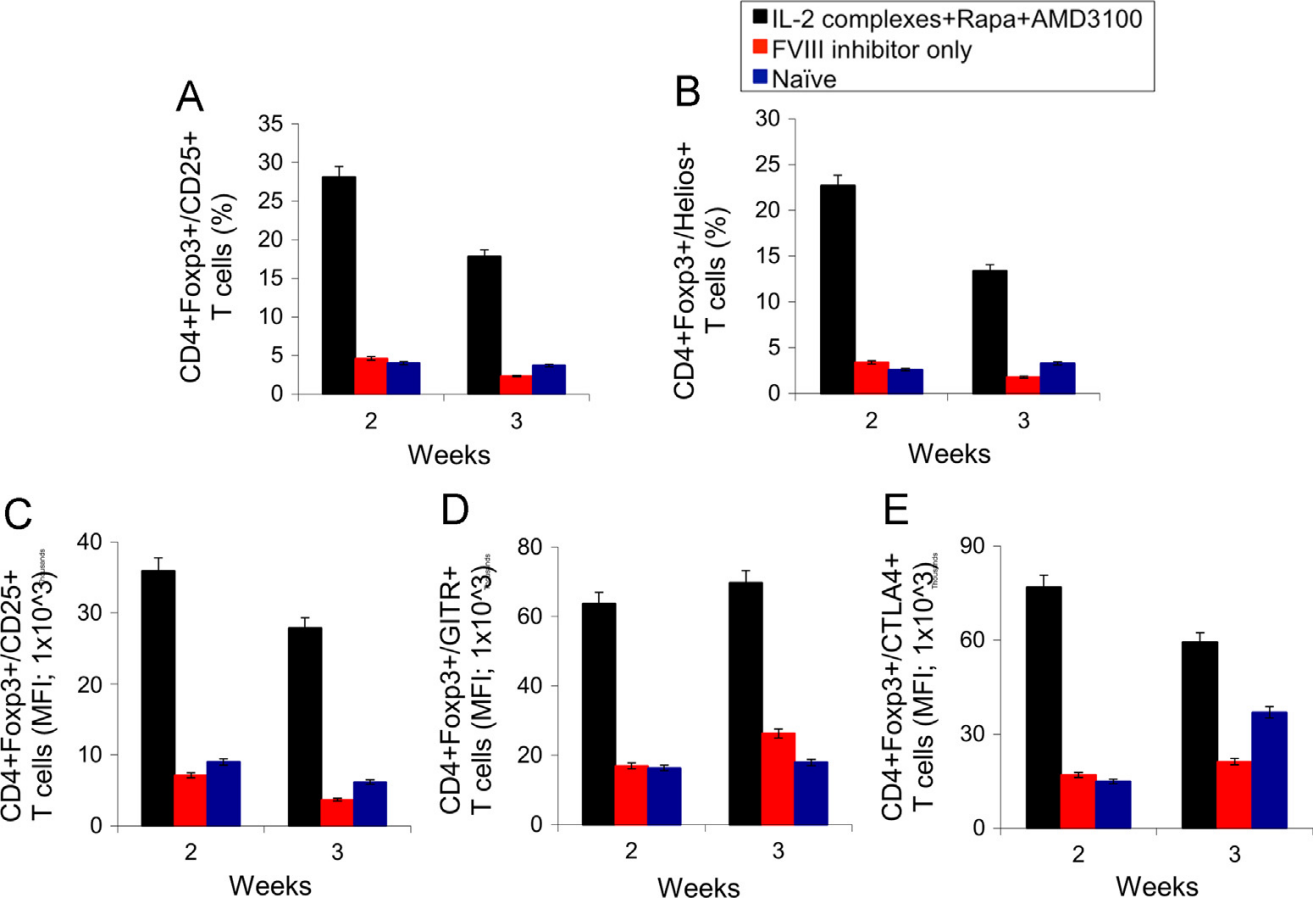
Effects of Immunomodulation on CD4^+^CD25^+^Foxp3^+^ and CD4^+^Foxp3^+^Helios^+^ Tregs, and Treg activation markers in peripheral blood in treated HemA inhibitor mice. PBMC were collected on weeks 2 and 3 after combination treatment (IL-2/IL-2mAb complexes+rapamycin+AMD3100) in HemA inhibitor mice (n=8), the percentage (%) of gated cells of CD4^+^CD25^+^Foxp3^+^ Tregs (A) and Helios^+^ Tregs (B) were investigated in each mouse group. MFI levels of Treg activation markers CD25^+^ (C), GITR^+^ (D) and CTLA4^+^ (E) were also investigated at different time points after treatment. Data shown is representative of two independent experiments.

**Fig. 5:**
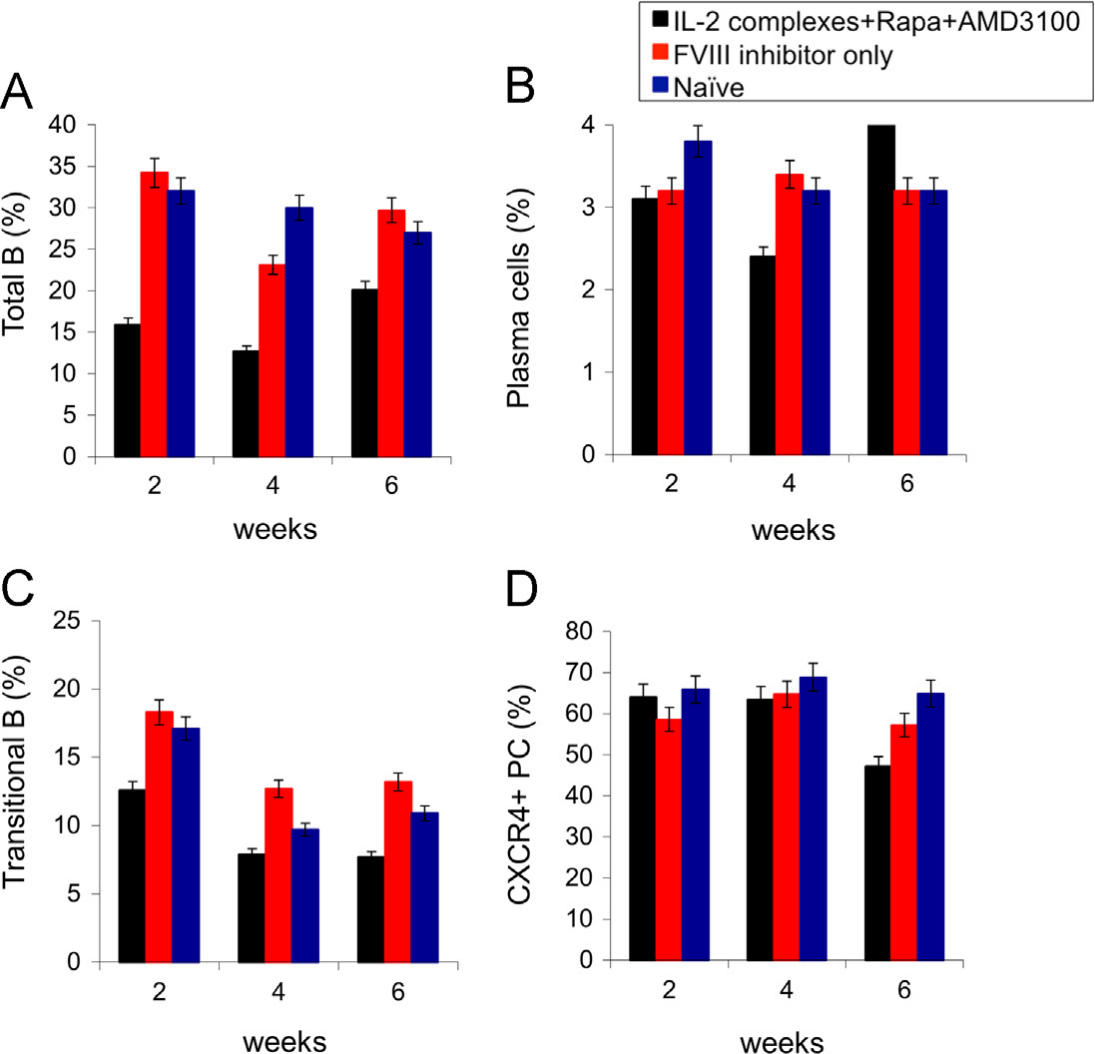
Effect of immunomodulation on total and transitional B cells, PCs and CXCR4^+^ PCs in peripheral blood of each mouse group (*n*=8). PBMCs were collected and stained for B cell and PC populations at different time points following treatment. (A) Total B cell (%), (B) PCs (%), (C) Transitional B cells (%) and (D) CXCR4^+^ PCs (%) were investigated using flow cytometry analysis. Data shown is representative of two independent experiments.

**Fig. 6:**
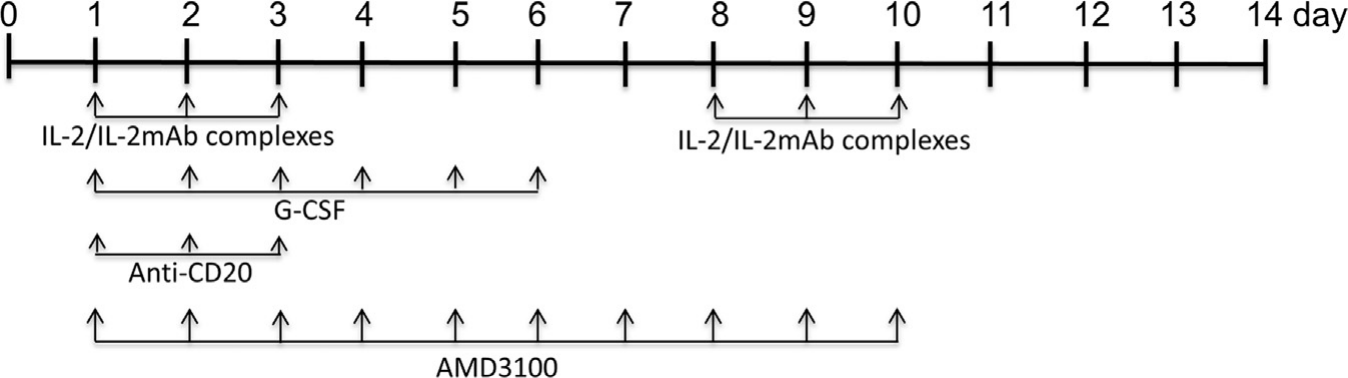
The combination treatment timeline for one cycle per two weeks. The combined regimens were given as shown in the timeline plotted. One cycle treatment includes two weeks long (14-days) period. For each cycle, IL-2/IL-2mAb complexes were given on Mon, Tue and Wed every week, G-CSF was administered everyday for 6-days in the first week, Anti-CD20 was given 3-days on Monday, Tuesday and Wednesday in the first week, and AMD3100 was administered everyday for 10-days within the 14-days treatment period.

**Fig. 7:**
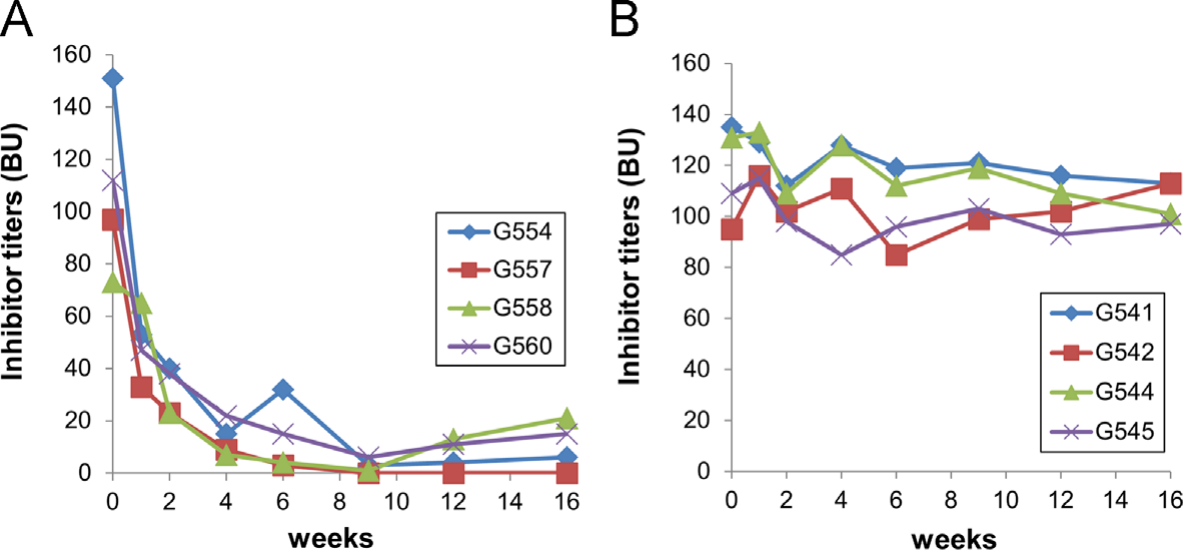
Inhibitor titers in *FVIII* plasmid primed HemA mice with pre-existing inhibitors following combination treatment. Two groups of HemA mice were treated separately with different combination regimens 2-weeks per cycle for 3 cycles: (A) IL-2/IL-2mAb complexes+anti-CD20+AMD3100+G-CSF (*n*=4), (B) Inhibitor mice only (*n*=4) as the control group. Peripheral blood was collected at different time points following the combination treatment. Anti-FVIII antibody titers were assessed by Bethesda assay over time. Each symbol represents data obtained from an individual mouse.

**Fig. 8:**
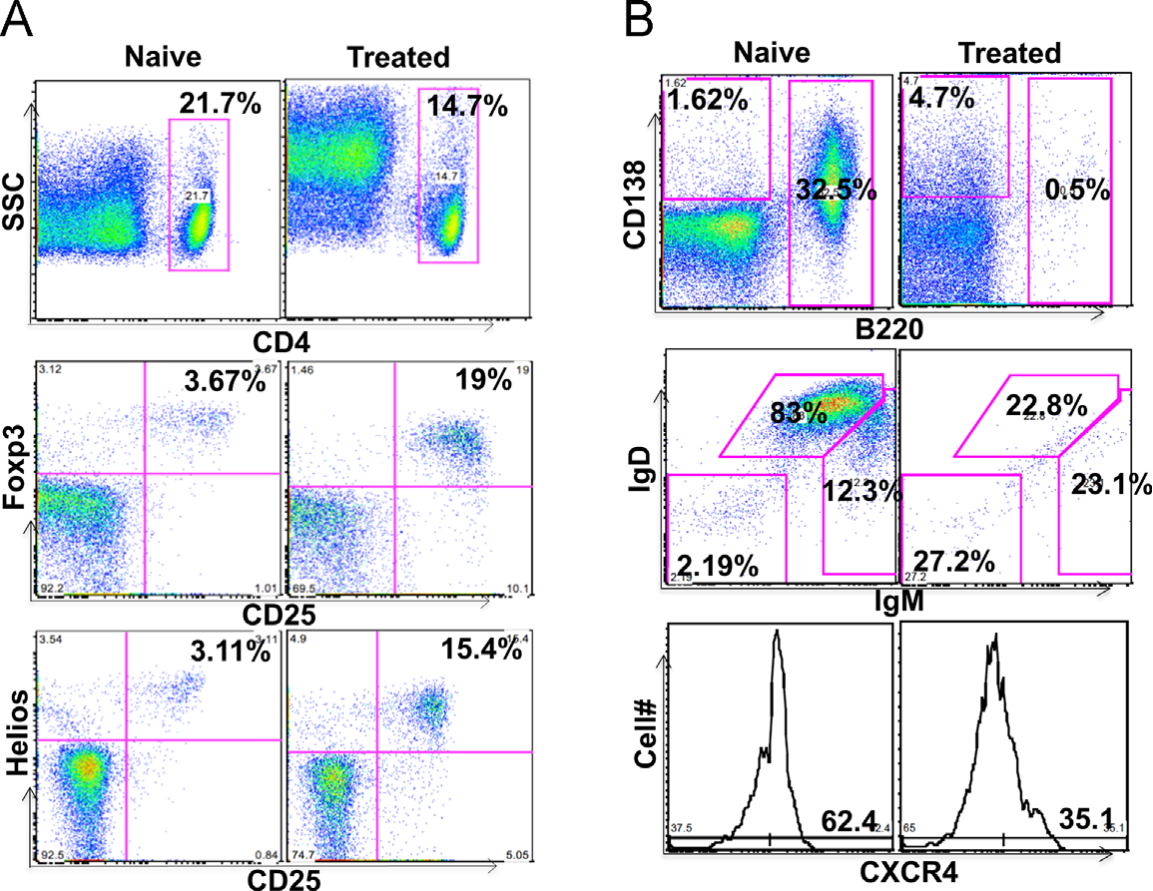
Gating strategies on CD4^+^CD25^+^Foxp3^+^, CD4^+^Foxp3^+^Helios^+^ Tregs, and B cell populations (total B, transitional B, PCs and CXCR4^+^PCs) in peripheral blood in treated HemA inhibitor mice. PBMCs were collected on indicated time points after combination treatment (IL-2/IL-2mAb complexes+G-CSF+AMD3100+anti-CD20) in HemA inhibitor mice. (A) The percentage (%) of gated cells of CD4^+^CD25^+^Foxp3^+^ Tregs and Helios^+^ Tregs were investigated in each mouse group. (B) Total B cell (%), PCs(%), transitional B cells (%) and CXCR4^+^ PCs (cell number) were gated using flow cytometry analysis.

**Fig. 9:**
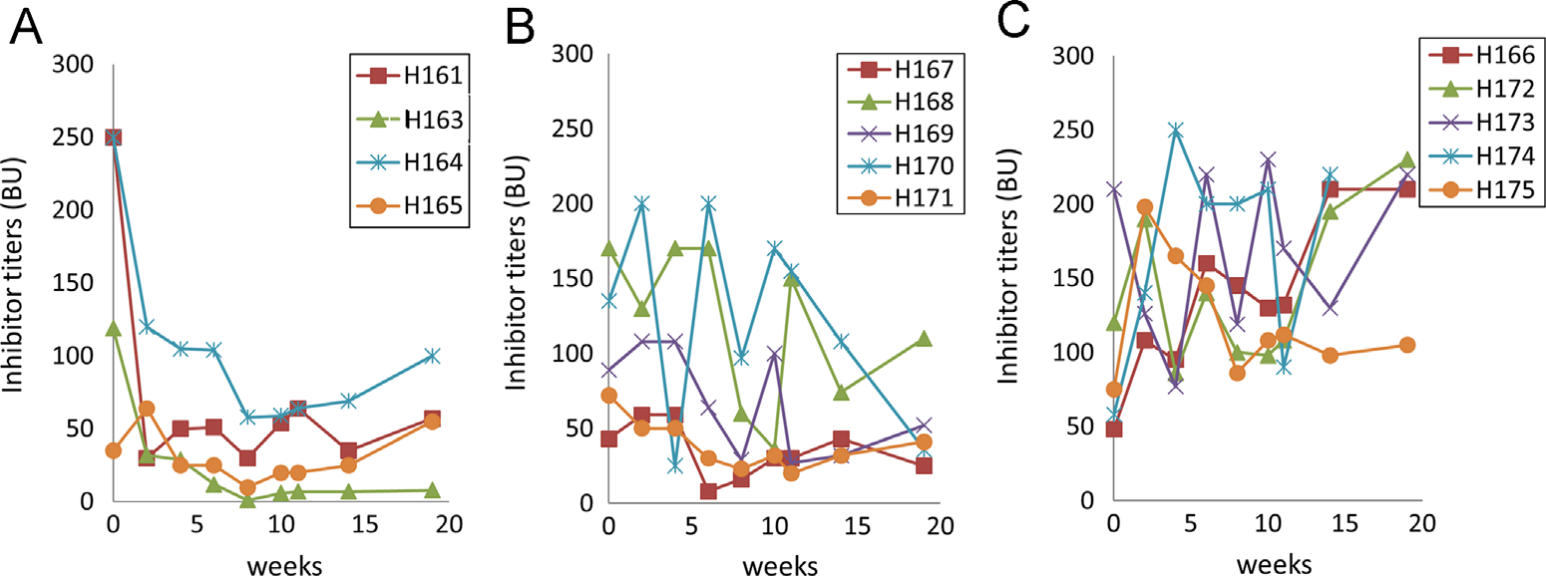
Inhibitor titers in FVIII protein primed HemA mice with pre-existing inhibitors following combination treatment. Two groups of HemA mice were treated separately with different combination regimens 2-weeks per cycle for 3 cycles: (A) IL-2/IL-2mAb complexes+anti-CD20+AMD3100+G-CSF (*n*=4), (B) Anti-CD20+AMD3100+G-CSF (*n*=5), and (C) Inhibitor mice only (*n*=5) as the control group. Peripheral blood was collected at different time points following the combination treatment. Anti-FVIII antibody titers were assessed by Bethesda assay over time. Each symbol represents data obtained from an individual mouse. Data shown is representative of two independent experiments.

Hemophilia A (HemA) mice were treated with either *FVIII* plasmid or protein to induce production of pre-existing inhibitory antibodies. These HemA inhibitor mice were then treated with either single agent or combination treatment. Immune responses against FVIII and FVIII activities were followed over time. Subsets of B and T cells were analyzed using flow cytometry. First, HemA inhibitor mice were treated with a single cycle (200 μg/mouse/day for 10 days) of AMD3100. HemA inhibitor mice were used as controls. Antibody titers in treated and control mice were shown in Fig. 1A and B. Staining results of total B and CXCR4^+^ plasma cells (PCs) were shown in Fig. 1C and D. Next, we explored the single cycle treatment of G-CSF (250 μg/kg/day for 5-days) in HemA inhibitor mice. Antibody titers and staining results of total B and PCs are shown in Fig. 2. Subsequently, we treated HemA mice with a combination regimen (IL-2/IL-2mAb complexes+rapamycin+AMD3100) for 4 weeks. Antibody titers in treated and control mice were shown in Fig. 3A and B. Peripheral blood mononuclear cells (PBMCs) in HemA mice were collected on weeks 2 and 3 following combination treatment and staining of CD4^+^CD25^+^Foxp3^+^and CD4^+^Foxp3^+^Helios^+^ Tregs, and Treg activation markers were carried out and analyzed by flow Cytometry (Fig. 4). In addition, total B cell (%; Fig. 5A), PCs (%; Fig. 5B) Transitional B cells (%; Fig. 5C) and CXCR4^+^PCs (%; Fig. 5D) were investigated using flow cytometry analysis. Furthermore, a new combination treatment was administered into HemA inhibitor mice. Fig. 6 demonstrates the combination treatment timeline for one cycle per every two weeks. The combination treatment include IL-2/IL-2mAb complexes + Anti-CD20+AMD3100+G-CSF. Inhibitor titers in *FVIII* plasmid primed HemA mice with pre-existing inhibitors following combination treatment and control HemA inhibitor mice over 16 weeks were shown in Fig. 7. A representative figure and gating strategies for characterizing CD4^+^CD25^+^Foxp3^+^, CD4^+^Foxp3^+^Helios^+^ Tregs, and B cell populations (total B, transitional B, PCs and CXCR4^+^PCs) in peripheral blood in treated HemA inhibitor mice was shown (Fig. 8). Inhibitor titers in FVIII protein primed HemA mice with pre-existing inhibitors following combination treatment over 18 weeks are shown in Fig. 9. The summary and interpretation of the data and results were described in Ref [1].

## 2 Experimental design, materials and methods

### 2.1 Mice

All mice were maintained at a specific pathogen-free (SPF) facility according to the National Institutes of Health guidelines for animal care and the guidelines of Seattle Children׳s Research Institute. HemA mice in a 129/SV×C57BL/6 mixed genetic background were used at the age of 6–8 weeks.

### 2.2 Generation of HemA inhibitor mice

We generated HemA inhibitor mice by injecting HemA mice intravenously (i.v.) with 50 μg of *FVIII* plasmid (pBS-HCRHPI-FVIIIA [2]) in 2 ml phosphate-buffered saline (PBS) via tail vein in 8–10 s, or intraperitoneally (i.p.) with low dose FVIII protein (2U/mouse/wk; Kogenate^®^, Bayer (Whippany, NJ)) consecutively for 4 weeks.

### 2.3 Administration of single or combined immunomodulating agents into mice

Immunomodulation agents with indicated dosages were administered into mice according to schedules specified in experiments. Blood samples were taken from the retro-orbital plexus at serial time points.

### 2.4 Flow cytometry and antibodies

Cell suspensions of peripheral blood and spleens of each treated mouse group were prepared according to standard protocols. Cell suspensions were stained for FACS analysis using appropriate antibodies. Samples were analyzed on an LSRII flow cytometer (Becton Dickinson, Palo Alto, CA) and data were analyzed using FlowJo software (Tree Star, Ashland, OR).

### 2.5 FVIII activities and inhibitor titers assays

Peripheral blood samples were taken from the experimental mice and collected in a 3.8% sodium citrate solution. FVIII activities were evaluated from the activated partial thromboplastin time (APTT) by a modified clotting assay using FVIII deficient plasma and reagents. FVIII activities were calculated from a standard curve generated with serially diluted normal human pooled plasma. Anti-FVIII antibody titers were measured by Bethesda assay.
