# Supplementary material for: The dataset from administration of single or combined immunomodulation agents to modulate anti-FVIII antibody responses in FVIII plasmid or protein primed hemophilia A mice
Source: Data Brief. 2016 Mar 17;7:973–80. doi: 10.1016/j.dib.2016.03.019 (PMC4818348; doi:10.1016/j.dib.2016.03.019)
Supplement: Supplementary material [file mmc1.pdf]

Title: The dataset from administration of single or combined immunomodulation agents to modulate anti-FVIII antibody responses in *FVIII* plasmid or protein primed hemophilia A mice

Authors: Chao Lien Liu[1, 2], Meghan J. Lyle[1], Simon C. Shin[1], Carol H. Miao[1,3,†]

Affiliations: [1] Center for Immunity and Immunotherapies, Seattle Children's Research Institute, Seattle, WA, USA

[2] School of Medical Laboratory Science and Biotechnology, College of Medical Science and Technology, Taipei Medical University, Taipei, Taiwan

[3] Department of Pediatrics, University of Washington, Seattle, WA, USA

Contact email: carol.miao@seattlechildrens.org

Conflicts of interest: none
